# Knowledge and skills of newborn resuscitation among health care professionals in East Africa. A systematic review and meta-analysis

**DOI:** 10.1371/journal.pone.0290737

**Published:** 2024-03-08

**Authors:** Gedefaye Nibret Mihretie, Tewachew Muche Liyeh, Alemu Degu Ayele, Bekalu Getnet Kassa, Habtamu Gebrehana Belay, Tigabu Munye Aytenew, Dagne Addisu Sewuye, Binyam Minuye Birhane, Agenesh Dereje Misk, Bekalu Kassie Alemu

**Affiliations:** 1 Department of Midwifery, College of Health Sciences, Debre Tabor University, Debre Tabor, Ethiopia; 2 School of Public Health, University of Technology Sydney, Sydney, Australia; 3 Department of Nursing, College of Health Sciences, Debre Tabor University, Debre Tabor, Ethiopia; 4 Department of Midwifery, Debre Markos University, Debre Markos, Ethiopia; Haramaya University Faculty of Health Sciences: Haramaya University College of Health and Medical Sciences, ETHIOPIA

## Abstract

**Introduction:**

Newborn resuscitation is a medical intervention to support the establishment of breathing and circulation in the immediate intrauterine life. It takes the lion’s share in reducing neonatal mortality and impairments. Healthcare providers’ knowledge and skills are the key determinants of the success of newborn resuscitation. Many primary studies have been conducted in various countries to examine the level of knowledge and skills of newborn resuscitation and associated factors among healthcare providers. However, these studies had great discrepancies and inconsistent results across East Africa. Hence, this review aimed to synthesize the pooled level of knowledge and skills of newborn resuscitation and associated factors among healthcare providers in East Africa.

**Method:**

Studies were systematically searched from February 11, 2023, to March 10, 2023, using PubMed, Google Scholar, HINARI, and grey literature. The effect size measurement of knowledge and skill of health care newborn resuscitation was estimated using the Random Effect Model. The data were extracted by Excel and analyzed using Stata 17 software. The Cochran’s Q test and *I*^*2*^ statistic were used to assess the heterogeneity of studies. The symmetry of the funnel plot and Egger’s test were used to check for publication bias. A subgroup analysis was done on the study years, sample sizes, and geographical location. Percentages and odds ratios (OR) with 95% CI were used to pool the effect measure.

**Results:**

In this systematic review and meta-analysis, a total of 1953 articles were retrieved from various databases and registers. Finally, 17 studies with 7655 participants were included. The overall levels of knowledge and skills of healthcare providers on newborn resuscitation were 58.74% (95% CI: 44.34%, 73.14%) and 46.20% (95% CI: 25.16%, 67.24%), respectively. Newborn resuscitation training (OR = 3.95, 95% CI: 2.82, 5.56) and the availability of newborn resuscitation guidelines (OR = 2.71, 95% CI: 1.90, 3.86) were factors significantly associated with knowledge of health care professionals on newborn resuscitation. Work experience (OR = 5.92, 95% CI, 2.10, 16.70), newborn resuscitation training (OR = 2.83, 95% CI, 1.8, 4.45), knowledge (OR = 3.05, 95% CI, 1.78, 5.30), and the availability of newborn resuscitation equipment (OR = 4.92, 95% CI, 2.80, 8.62) were determinant factors of skills of health care professionals on newborn resuscitation.

**Conclusion:**

The knowledge and skills of healthcare providers on newborn resuscitation in East Africa were not adequate. Newborn resuscitation training and the availability of resuscitation guidelines were determinant factors of knowledge, whereas work experience, knowledge, and the availability of newborn resuscitation equipment and training were associated with the skills of healthcare providers in newborn resuscitation. Newborn resuscitation training, resuscitation guidelines and equipment availability, and work experience are recommended to improve healthcare providers’ knowledge and skills.

## Introduction

Newborn resuscitation is a component of essential newborn care that focuses on breathing stimulation and ventilation with a bag and mask. The first 10 minutes after birth are the golden minutes for newborn resuscitation [1]. Neonatal resuscitation is a technique for reviving an asphyxiated baby, but it is more challenging than adult resuscitation. Basic newborn resuscitation includes airway clearing, head positioning, and positive pressure ventilation with a bag and masks medication, and chest compression [2,3].

The neonatal period is the most vulnerable and high-risk period in the lives of neonates because the highest rates of mortality and morbidity occur during this period [4–6]. Every year, closely four million babies worldwide die within the first month of life, with approximately 99% of these deaths occurring in low- and middle-income countries [7]. About 1.2 million African babies died in the first four weeks of life, with half of them dying within the first 24 hours after delivery [8]. According to the World Health Organization, developing countries including the Horn of Africa, account for 99% of the 3.8 million newborn mortality [9]. According to the 2019 mini Ethiopia Demographic and Health Surveys, Ethiopia has a high neonatal mortality rate of 33 per 1,000 live births (1 in 33 children), with a slight increase over the past few years [10].

Perinatal asphyxia is the leading cause of neonatal death and is responsible for 23% of newborn mortality, and it is still a global problem [5,11,12]. Every newborn should be considered at risk for birth asphyxia during birth because the majority of causes are unpredictable [13,14]. Socioeconomic factors, demographics, the healthcare system, maternal health before, during, and after pregnancy, delivery practices, cultural practices, and postnatal care of babies are all direct and indirect determinants of neonatal mortality [15–17]. The World Health Organization’s newborn resuscitation guidelines indicate that if a newborn is resuscitated quickly and efficiently and begins breathing spontaneously within 20 minutes of birth, the risk of sequelae is low. But the success of resuscitation is depending on knowledge, preparation, and skills of healthcare providers, functional equipment, a timely beginning, and correct procedures [18].

Reduction of neonatal mortality is world health policy agenda since the Millennium Development Goal cannot be achieved without significant reductions in neonatal mortality [19]. The World Health Assembly and Sustainable Development Goals endorsed a road map for preventable newborn deaths and stillbirths, requiring all countries to achieve 12 or fewer neonatal deaths per 1,000 live births by 2030 [20,21]. According to a Global Development Alliance report, out of 139 million newborns, 17 million require resuscitation to breathe, and nearly 700,000 die due to birth asphyxia [22]. Globally, poor resuscitation practice remains one of the leading causes of neonatal mortality and morbidity [6,23–25].

Newborn resuscitation is a proven clinical practice that reduces intrapartum-related neonatal asphyxia and its complications [26–28]. In addition, newborn resuscitation contributes to a significant reduction in neonatal mortalities and morbidities due to birth asphyxia [29–31]. The success of newborn resuscitation is affected by the healthcare provider’s knowledge, skills, and availability of neonatal resuscitation equipment [32]. Other studies indicated that healthcare workers must be knowledgeable and skilled in newborn resuscitation, and they must screen neonates for possible complications early and take immediate action [33,34]. Educational level, work experience, newborn resuscitation training, accessibility of resuscitation guidelines and equipment, and knowledge of newborn resuscitation were some factors in healthcare providers’ knowledge and skills in neonatal resuscitation [11,35,36].

Practicing neonatal resuscitation has shown a significant reduction in intrapartum stillbirths and first-day neonatal mortality [37]. Many primary studies have been conducted in various countries to examine the prevalence of knowledge and skills of newborn resuscitation and associated factors among healthcare providers. However, these studies had great discrepancies and inconsistent results. The prevalence of knowledge and skills of newborn resuscitation ranged from 9.8% [7] to 92% [38], and 11.7% [39] to 89.2% [36], respectively, across East Africa. Hence, this study included studies conducted in the last ten years (from 2013 to 2023) and synthesized the pooled prevalence of the knowledge and skills of newborn resuscitation and their determinant factors among healthcare providers in East Africa. Identifying the overall prevalence and factors of knowledge and skill of newborn resuscitation will help to formulate preventive strategies and management of perinatal asphyxia and its complications.

## Methods

### Study design and settings

This systematic review and meta-analysis was conducted in East Africa (Ethiopia, Kenya, Somalia, Eritrea, Djibouti, Tanzania, Uganda, Rwanda, Burundi, South Sudan, and Sudan). This study was registered as a protocol in the International Prospective Register of Systematic Reviews (PROSPERO ID: CRD42023400808).

### Data source and search strategy

This review and meta-analysis were developed based on the PRISMA (Preferred Reporting Items for Systematic Review and Meta-Analysis) guidelines [40]. Studies about knowledge and skills of newborn resuscitation were identified through an online search of PubMed, HINARI, and Google Scholar (**S1 File**). The studies were searched from February 11, 2023, to March 15, 2023.

### Eligibility criteria (based on “PPEOLD”)

**Inclusion criteria. Population:** The study participants were healthcare providers (including nurses, midwives, general practitioners, residents, pediatricians, and obstetric gynecologists) who work in the labour and delivery ward, operation room technique, and neonatal intensive care unit.

**Publication year:** The included studies were conducted from January 1, 2013, to January 1, 2023.

**Exposure:** factors associated with knowledge and skills of newborn resuscitation and at least two times reported as significant factors (newborn resuscitation training, work experience, availability of resuscitation equipment, knowledge of newborn resuscitation, and availability of newborn resuscitation guidelines in the health facilities).

**Outcome measurement:** knowledge and skills of newborn resuscitation among health care providers in East Africa (Table 1). Those who scored more than or equal to 80% of knowledge and skill assessment questions were considered to have good knowledge and good skills. Newborn resuscitation includes airway clearing, head positioning, and positive pressure ventilation with a bag and masks, medication, and chest compression.

**Language**: All included studies were reported in the English language.

**Design**: Published case-control and cross-sectional studies were included.

**Exclusion criteria:** Citations without full texts, duplicate studies, anonymous reports, pre- and post-newborn resuscitation training reports, and qualitative studies were excluded.

**Table 1:** Population Exposure Comparison and the Outcome variable (PECO) summary table.

Population	Exposure	Comparison		Outcomes (2013 to 2023)
Health care providers	Training	A healthcare provider who had taken newborn resuscitation training	*Healthcare providers who did not have taken training	Knowledge and skills in newborn resuscitation
Health care providers	Availability of resuscitation equipment	health facilities had resuscitation equipment	*Health facilities had no resuscitation equipment	Knowledge and skills in newborn resuscitation
Health care providers	Knowledge of newborn resuscitation	Participants who had good knowledge of newborn resuscitation	*Participants who had poor knowledge of newborn resuscitation	Skills of newborn resuscitation
Health care providers	Work experience	Healthcare providers who had work experience of more than one year	*Healthcare providers who had work experience of less than one year	Skills of newborn resuscitation
Health care providers	Availability of newborn resuscitation guidelines	Health facilities that had resuscitation guideline	* Health facilities that had no resuscitation guideline	Skills of newborn resuscitation

* = Reference Group.

### Screening and data extraction

Articles were searched, screened, and extracted by all 10 authors independently. The articles were screened by carefully reading the titles, abstracts, and the whole manuscript. Full-text articles were exported to the Endnote X7 reference manager software, and duplicated articles were excluded. Then, the full text of the studies was further evaluated based on objectives, methods, population, and outcomes, and then extracted all necessary data using a standardized data extraction form. The form includes the primary author, study year, publication year, study setting, sample size, study design, population, exposure, outcome, and specific factors associated with knowledge and skills of newborn resuscitation. Independent variables must be at least two times reported as significant factors.

### Quality assessment

The Newcastle-Ottawa Scale quality assessment tool designed for quality assessments was used to assess the scientific strength and quality of each primary study [41]. Using the assessment tool, the two authors independently weighted the quality of each original study. An assessment with a score of 50% or above was included in the analysis (5 out of 10). Score discrepancies between investigators were managed by taking the average scores (**S2 File**).

### Publication bias and heterogeneity

To reduce the risk of bias, extensive database, and manual searching were done. The authors’ cooperative work in the selection of articles based on clear objectives and eligibility criteria was also crucial in reducing publication bias. We examine publication bias with a visual inspection of the funnel plot graph asymmetry. Besides, Egger’s correlation tests at a 5% significant level were done to assess the presence of publication biases [42,43]. Furthermore, to identify the random variations among the primary studies point estimates and subgroup analysis were done. Heterogeneities across the studies were evaluated using *I*^*2*^ statistics with their corresponding p-value.

#### Statistical analysis and presentation

The result was summarized by pooled prevalence and odd ratio. We used STATA-17 software for analysis. The random-effects model was used to assess the effect size of the studies. The results were presented using texts, tables, and forest plots with a 95% confidence interval.

### Ethics approval and consent to participate

Not applicable.

## Results

### Study selection

**Fig 1:**
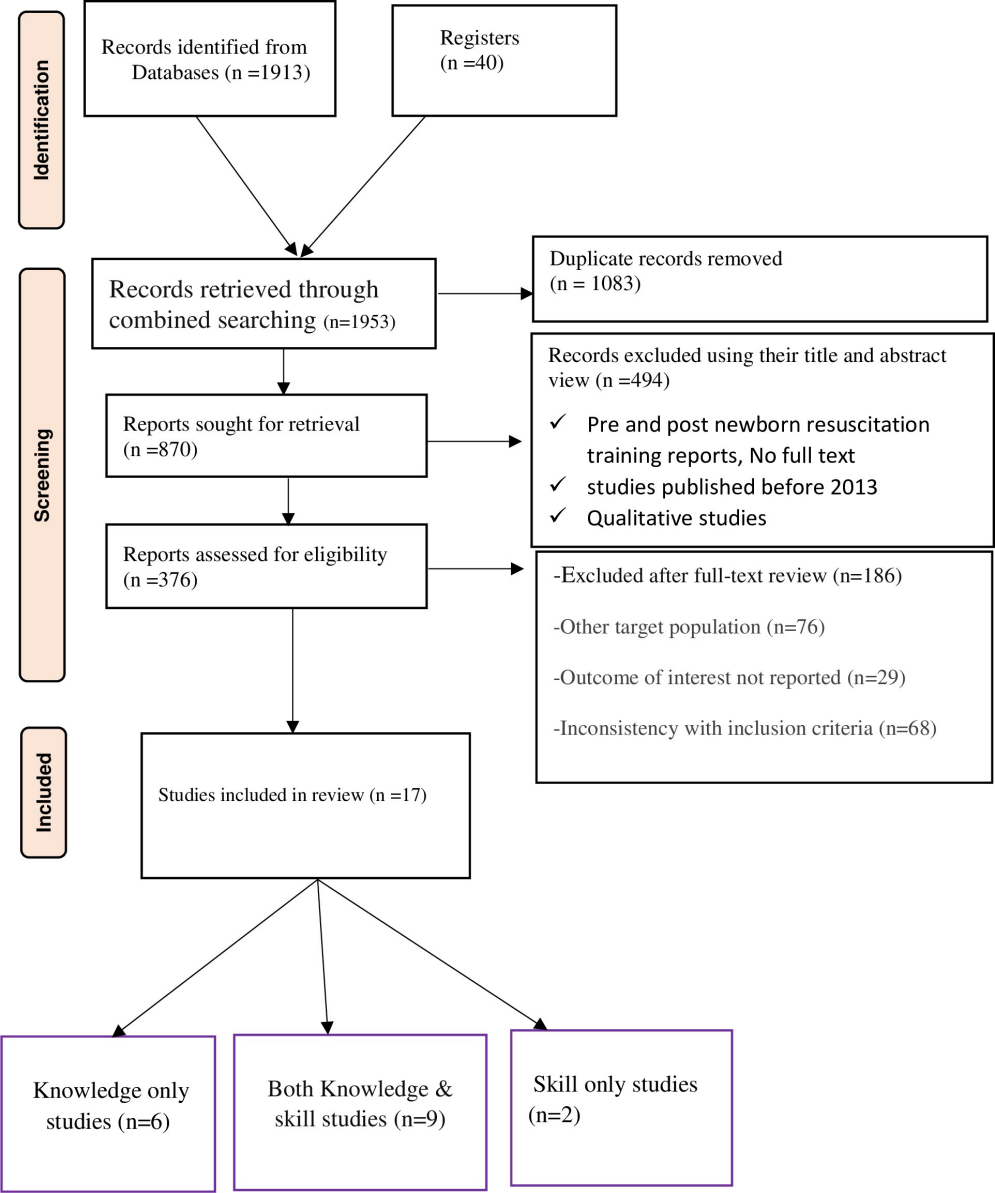
PRISMA flow chart revealing the identification of knowledge and skills of newborn resuscitation studies among health professionals via databases and registers in East Africa.

Using the medical, and health electronic databases and other relevant sources, 1953 records were identified and of these, 1083 were excluded due to duplication, and 494 articles were excluded after reviewing the title and the abstract. Of the remaining 476 articles, 457 were excluded due to different outcomes of interest, different operational definition measurements, other target populations, outcomes of interest not reported, and inconsistency with inclusion criteria. Finally, 17 studies were included in the systematic review and meta-analysis with 7655 participants. Among the 17 included studies, 9 were both knowledge and skill of newborn resuscitation articles; 6 were only knowledge studies, and 2 were only newborn resuscitation skills (**Fig 1**).

### Description of the included studies

The 17 eligible studies were case-control and cross-sectional studies in design. The sample sizes ranged from 43 in Sudan [44] to 3804 in Ethiopia [45]. Regarding the geographical distribution of studies, from Ethiopia(n = 8) [7,22,36,45–49], Kenya (n = 3) [50–52], Sudan (n = 1) [44], Tanzania (n = 3) [53–55], Somalia (n = 1) [56], and Uganda(n = 1) [38]. All these studies dealt with knowledge and skills of newborn resuscitation among healthcare providers in East African countries (**Table 2**).

**Table 2:** Description of included studies reporting the knowledge and skills of newborn resuscitation among health care providers in East Africa, 2013 to 2023.

First Author & Year of Study	Country	Study design	Participant	Sample size	Response rate	Prevalence of knowledge of NR	Prevalence of skills of NR	Outcome measurement	Quality
Gebreegziabher E et al. 2014[46]	Ethiopia	Cross-sectional	Health care providers	150	90%	66.3%	40%	Knowledge & skills of newborn resuscitation	8(good)
Sintayehu Y et al., 2020[7]	Ethiopia	Cross-sectional	Health care providers	427	96.6%	9.8%	11.7%	Knowledge & skills of newborn resuscitation	8(good)
Abrha MW et al., 2019[45]	Ethiopia	Cross-sectional	Health care providers	3804	100%	49.72%	89.2%	Knowledge of newborn resuscitation	9 (very good)
Bogale M et al., 2021[47]	Ethiopia	Cross-sectional	Health care providers	180	95.5%	89.0%	-	Knowledge of newborn resuscitation	7(good)
Bizuwork K et al., 2019[22]	Ethiopia	Cross-sectional	Health care providers	143	100	32.9%	24.5%	Knowledge & skills of newborn resuscitation	8(good)
Bekele, F.A et al., 2021[48]	Ethiopia	Cross-sectional	Health care providers	360	100%	46.5%	-	Knowledge of newborn resuscitation	8(good)
Abebaw M et al., 2022[36]	Ethiopia	Cross-sectional	Health care providers	409	100%	87.3%	89.2%	Knowledge & skills of newborn resuscitation	8(good)
Mersha A et al., 2020[49]	Ethiopia	Cross-sectional	Health care providers	445	96.4%	76.2%	71.1%	Knowledge & skills of newborn resuscitation	8(good)
MULI DM., 2020[50]	Kenya	Cross-sectional	Health care providers	201	100%	56.0%	41.0%	Knowledge & skills of newborn resuscitation	8(good)
Carolyne K et al, 2019[51]	Kenya	Cross-sectional	Health care providers	98	100%	78.0%	44.0%	Knowledge & skills of newborn resuscitation	8(good)
Kamau PT et al.,2022[52]	Kenya	Cross-sectional	Health care providers	46	100%	-	46.0%	The skill of newborn resuscitation	7(good)
Ahmed MA et al., 2022[56]	Somalia	Cross-sectional	Health care providers	98	100%	42.5%	-	Knowledge of newborn resuscitation	8(good)
Adlan YA et al, 2020[44]	Sudan	Cross-sectional	Health care providers	43	100%	41.0%	-	Knowledge of newborn resuscitation	7(good)
Joho AA et al., 2020[53]	Tanzania	Cross-sectional	Health care providers	176	98.0%	53.9%	53.2%	Knowledge & skills of newborn resuscitation	7(good)
Mzurikwao CB et al. 2018[54]	Tanzania	Case control	Health care providers	330	100%	-	-	Knowledge & skills of newborn resuscitation	8(good)
Mbinda MA et al., 2022[55]	Tanzania	Cross-sectional	Health care providers	340	100%	-	41.0%	The skill of newborn resuscitation	8(good)
Namuguzi M et al., 2020[38]	Uganda	Cross-sectional	Health care providers	75	100%	92.0%	-	Knowledge of newborn resuscitation	8(good)

NR = newborn resuscitation.

### Meta-analysis

#### Knowledge of healthcare providers on newborn resuscitation

The pooled prevalence of knowledge of newborn resuscitation among healthcare providers in East Africa was 58.74% (95% CI: 44.34%,73.14%) p = 0.0001, *I*^*2*^ = 99.34% [7,22,36,38,44–51,53,56].

#### Skills of healthcare providers on newborn resuscitation

The overall estimate of newborn resuscitation skills among healthcare providers was 46.20% (95% CI: 25.16%, 67.24%) [7,22,36,46,49–53,55].

#### Publication bias

**Fig 2:**
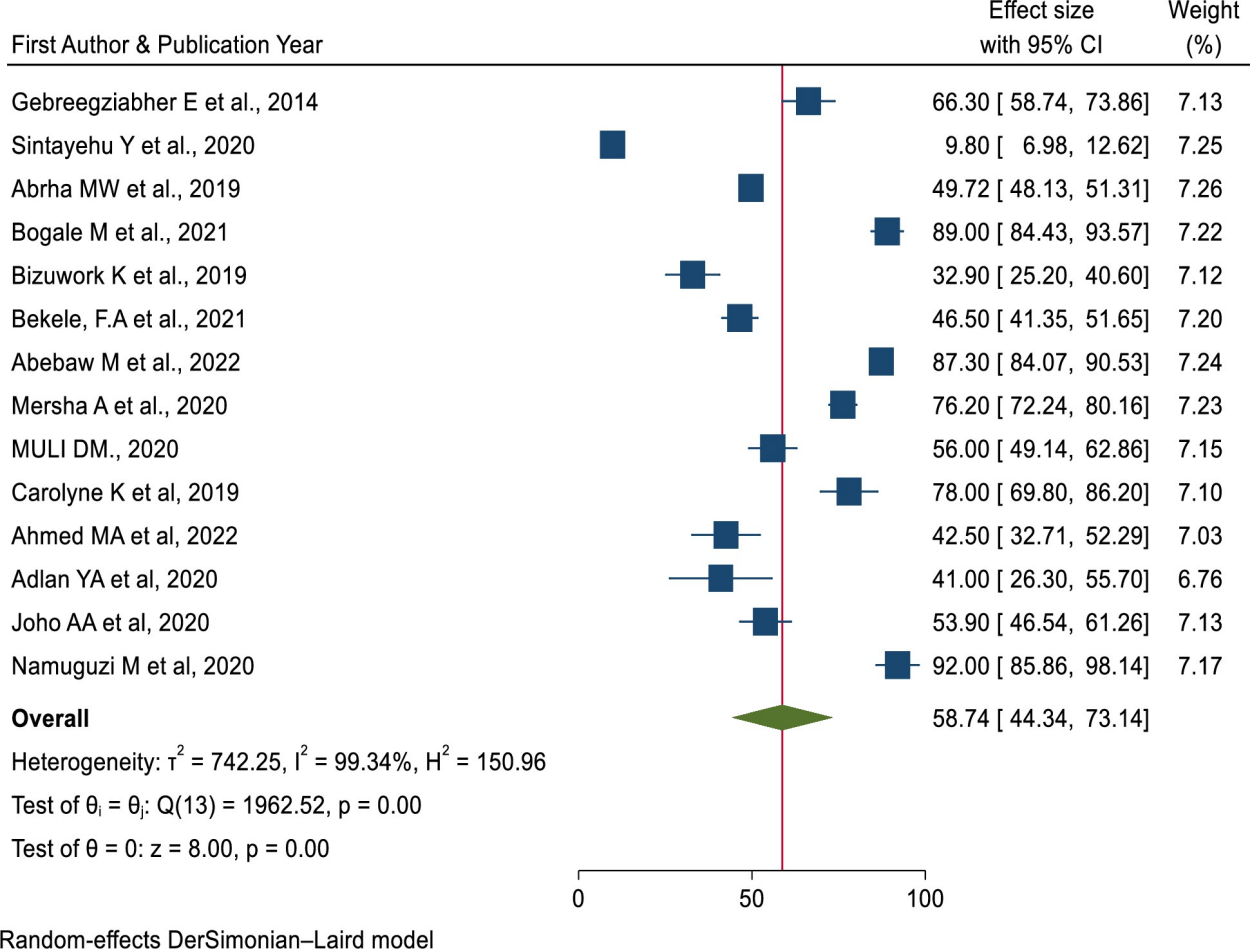
Funnel plot for assessing publication bias in the knowledge of health care providers on newborn resuscitation, East Africa, 2023.

**Fig 3:**
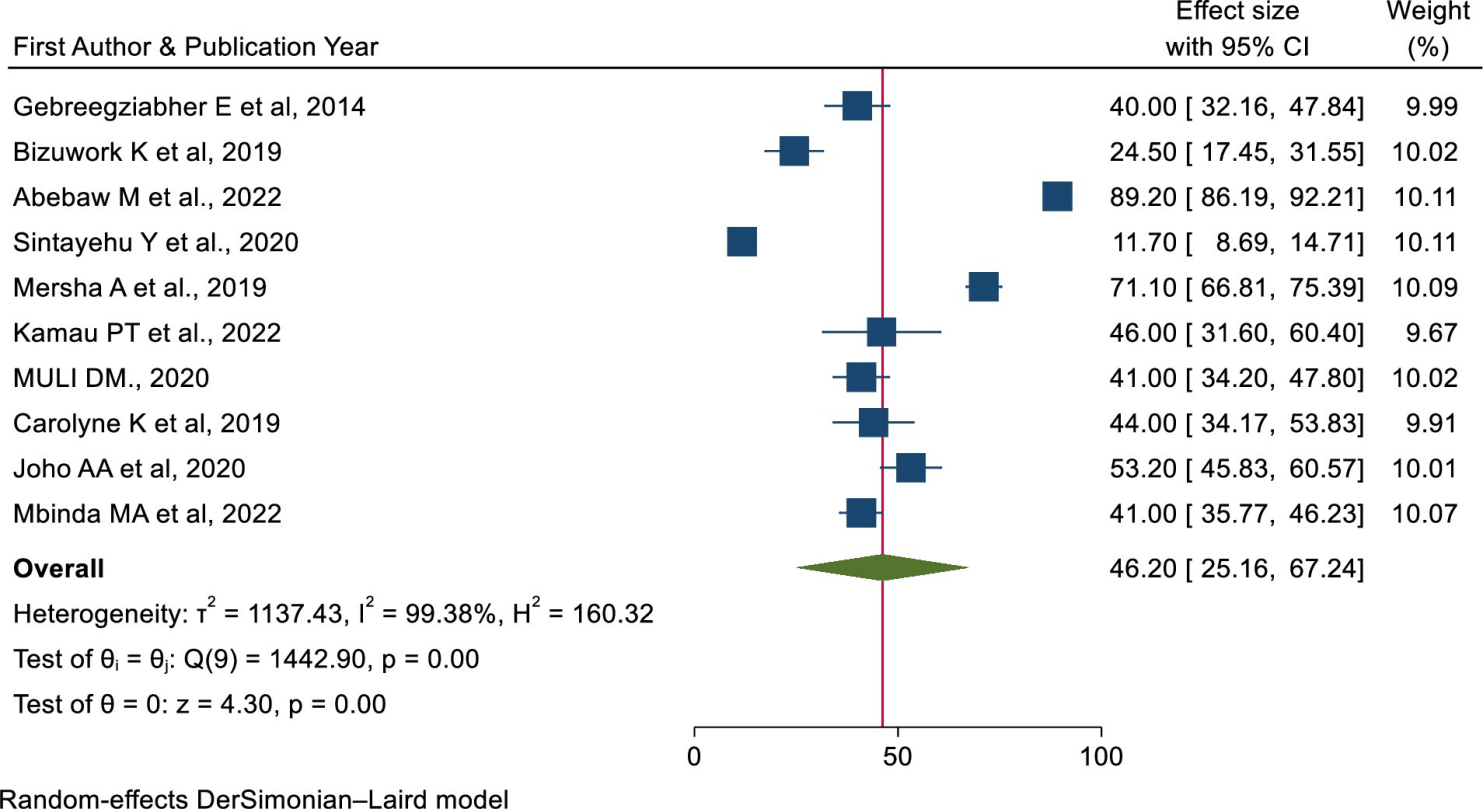
Funnel plot for assessing publication bias in the skills of health care providers on newborn resuscitation, East Africa, 2023.

The publication biases of knowledge-assessed studies were assessed using funnel plots and Egger’s regression test. The funnel plot results were likely to be asymmetric (**Fig 2**), but Egger’s regression test revealed no indication of publication bias across studies (P = 0.662). Publication biases in skill-assessed studies were also evaluated using funnel plots and Egger’s regression test. The funnel plot results revealed an asymmetric shape, indicating the presence of publishing bias (**Fig 3**). Egger’s regression test also revealed that there was publication bias across studies (P< 0.001).

**Fig 4:**
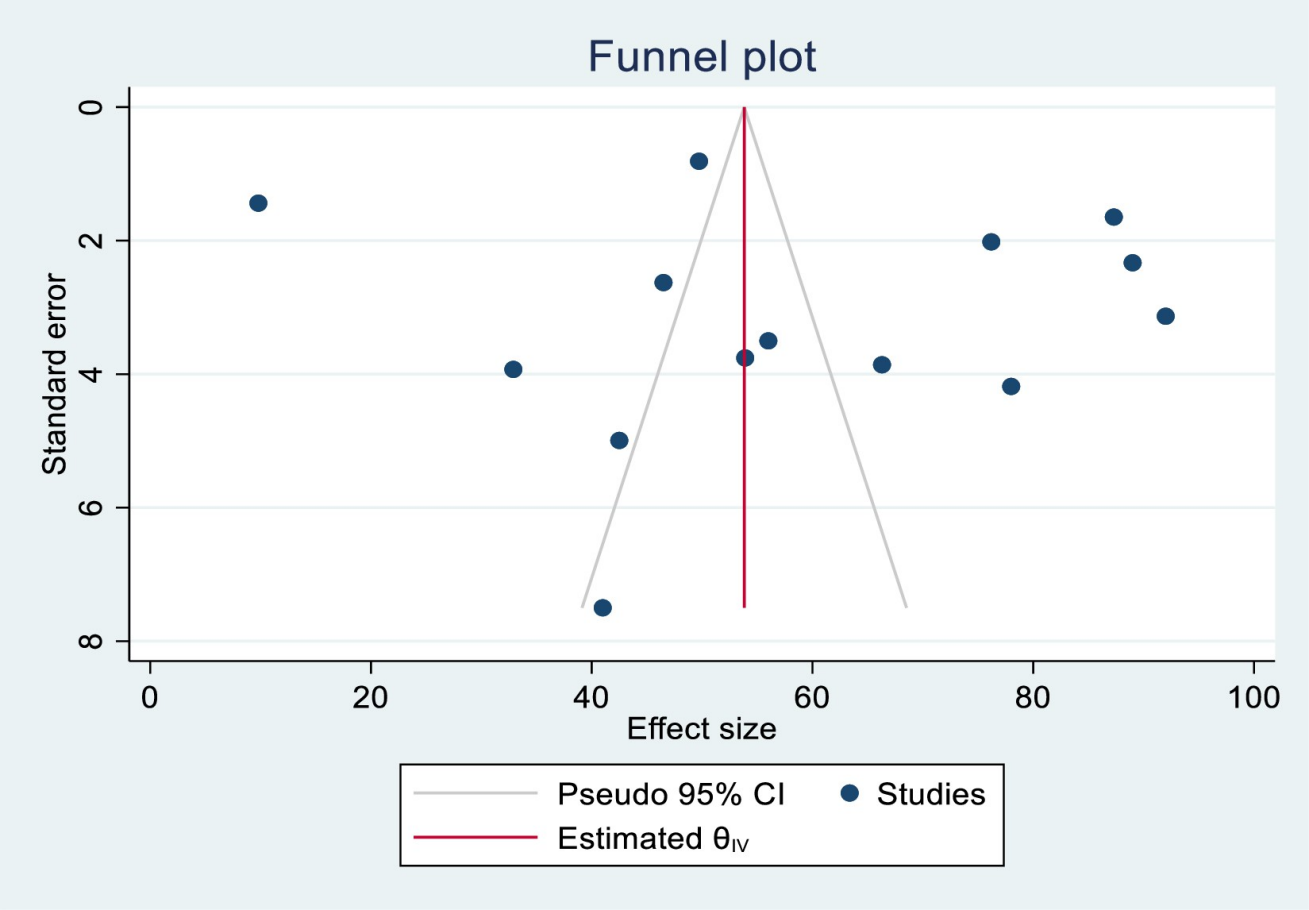
The funnel plots showed the results of the trim and fill analysis for adjusting publication bias in the 13 studies addressing newborn resuscitation skill.

Nonparametric trim and fill analyses were done among the newborn resuscitation skill assessment studies. Publication bias was corrected when three missed studies were imputed to the right side by trimming and filling analysis. After imputed 3 studies, 13 studies were included and resulting in a pooled prevalence of 55.23% (95% CI: 35.85%, 74.60%) (**Fig 4**).

**Fig 5:**
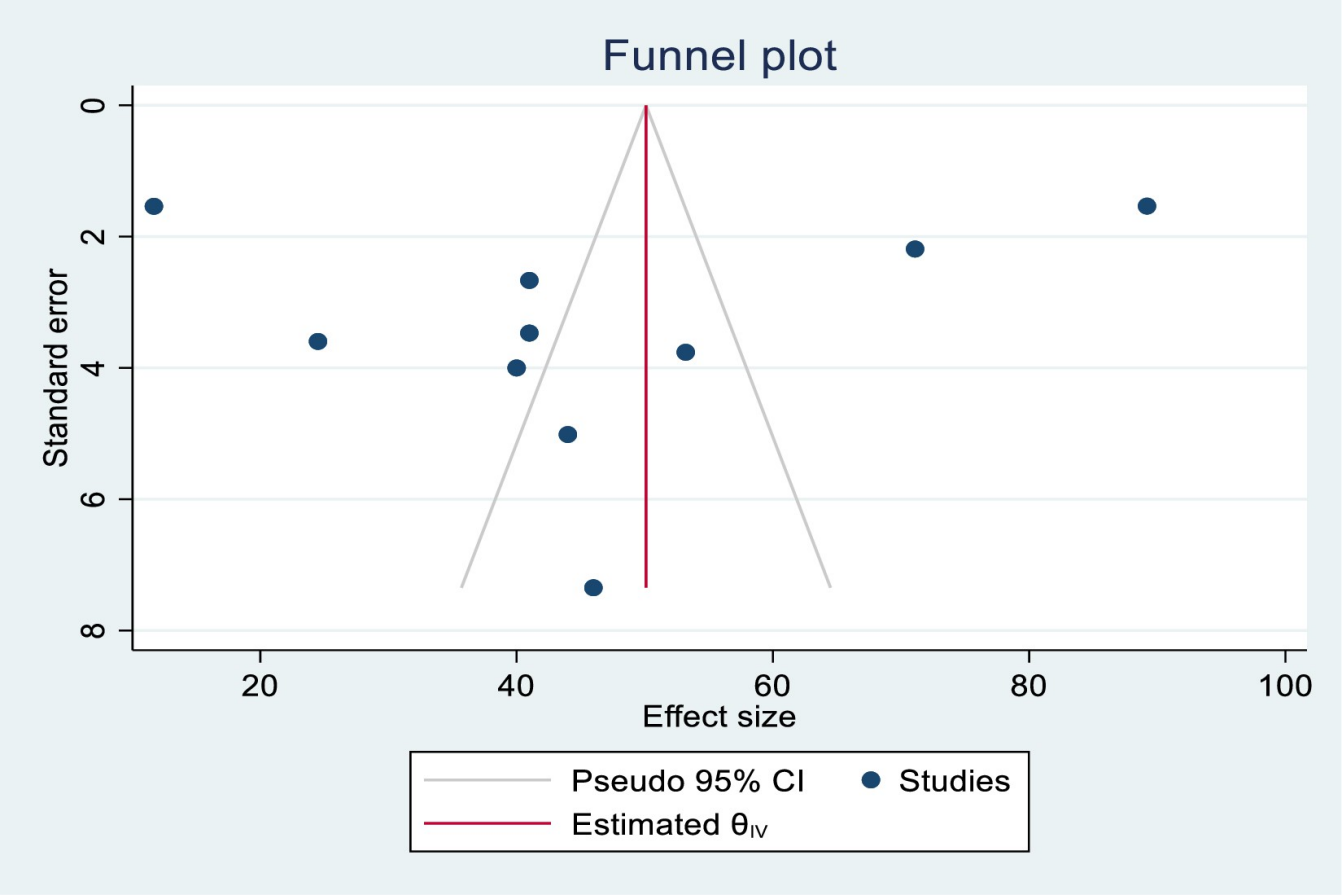
Forest plot of the pooled prevalence of Knowledge of neonatal resuscitation among health care providers in East Africa.

**Fig 6:**
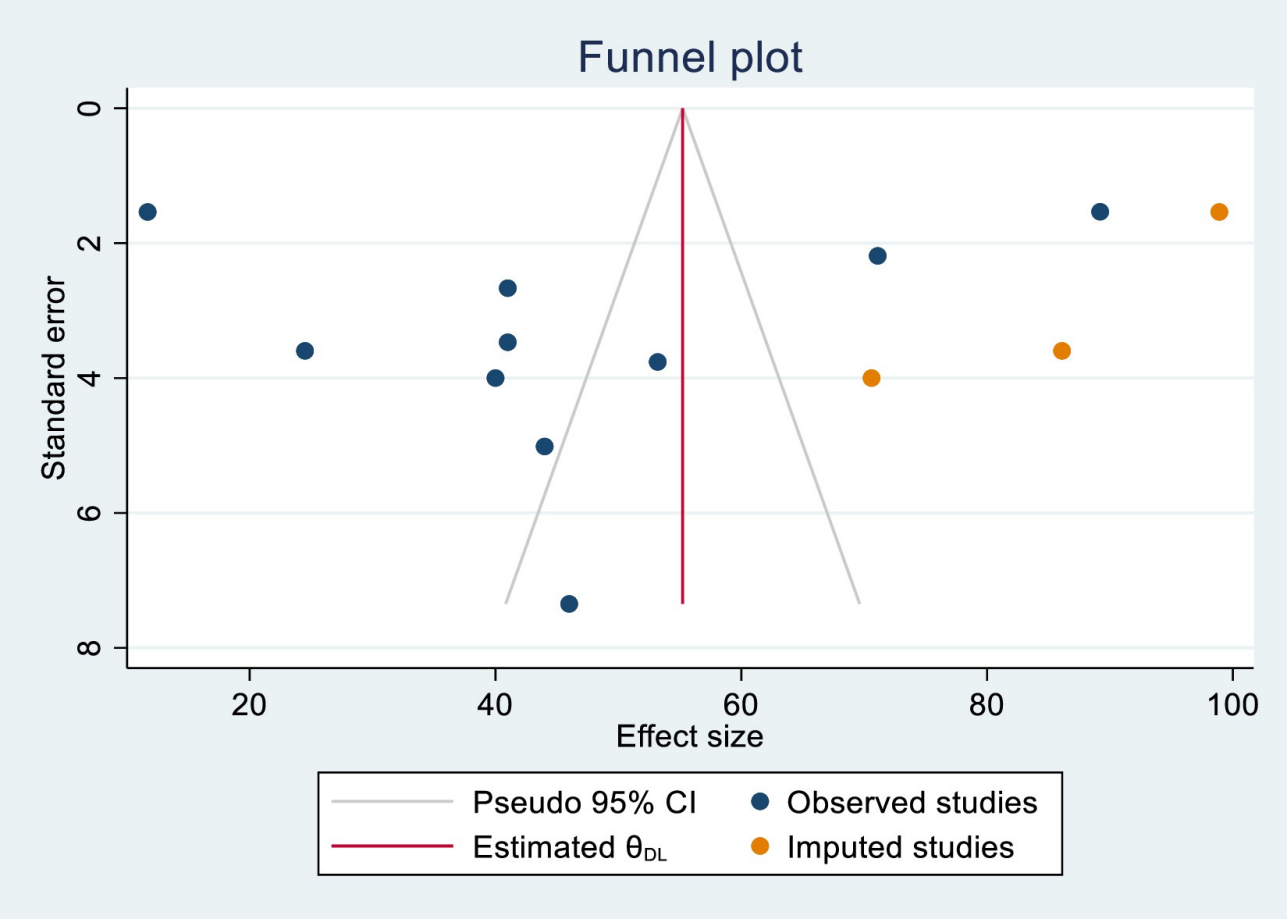
Forest plot of the pooled prevalence of skills of neonatal resuscitation among health care providers in East Africa.

*Heterogeneity*. The heterogeneity of knowledge and skills of newborn resuscitation among healthcare providers were *I*^*2*^ *= 99*.*34% (****Fig 5****) and I*^*2*^ *= 99*.*38%*r **(Fig 6).**

### Examine the source of heterogeneity

#### Knowledge of newborn resuscitation subgroup analysis

**Fig 7:**
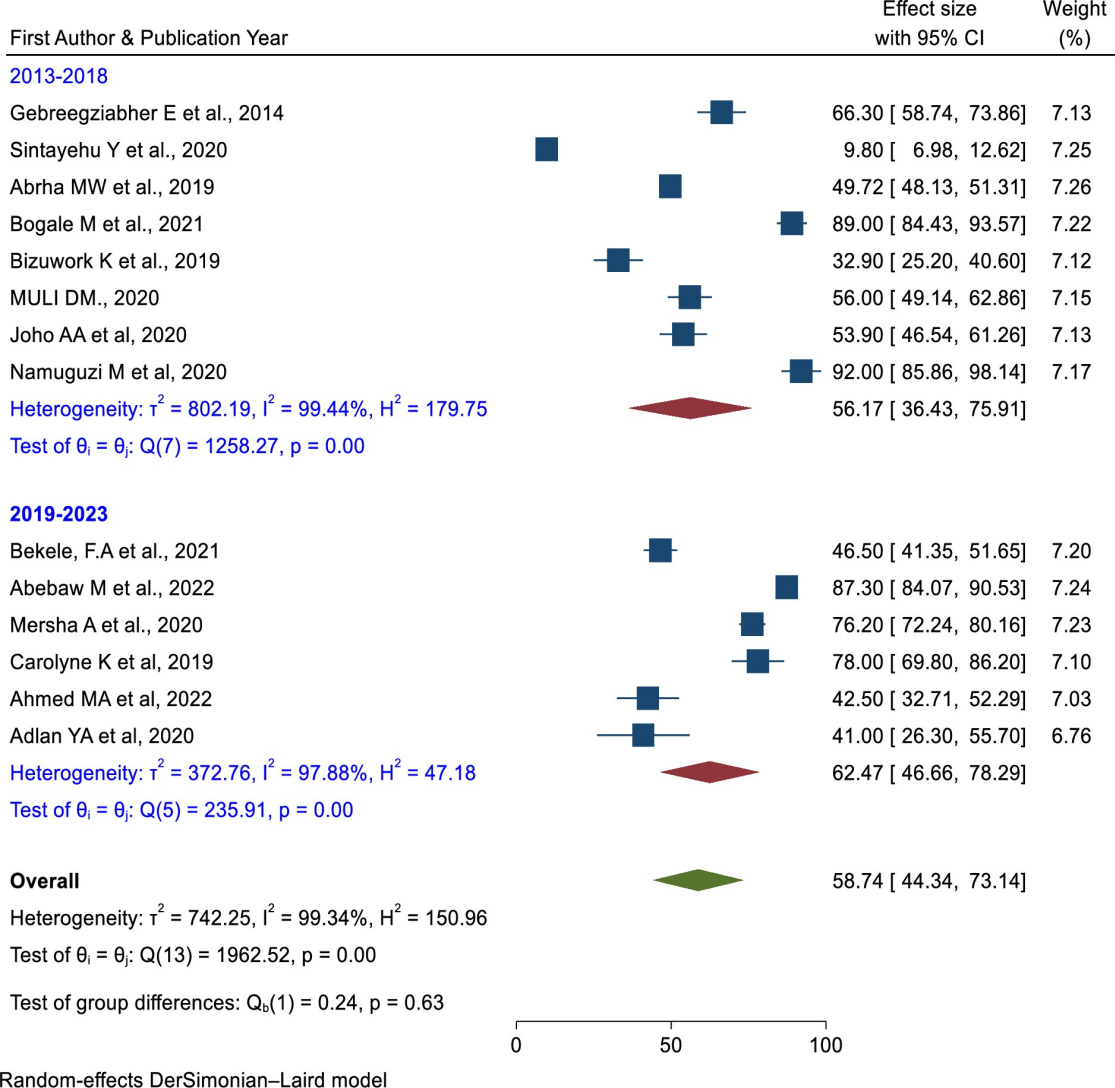
Subgroup analysis of the pooled prevalence of knowledge of newborn resuscitation among healthcare providers based on the study period in East Africa.

**Fig 8:**
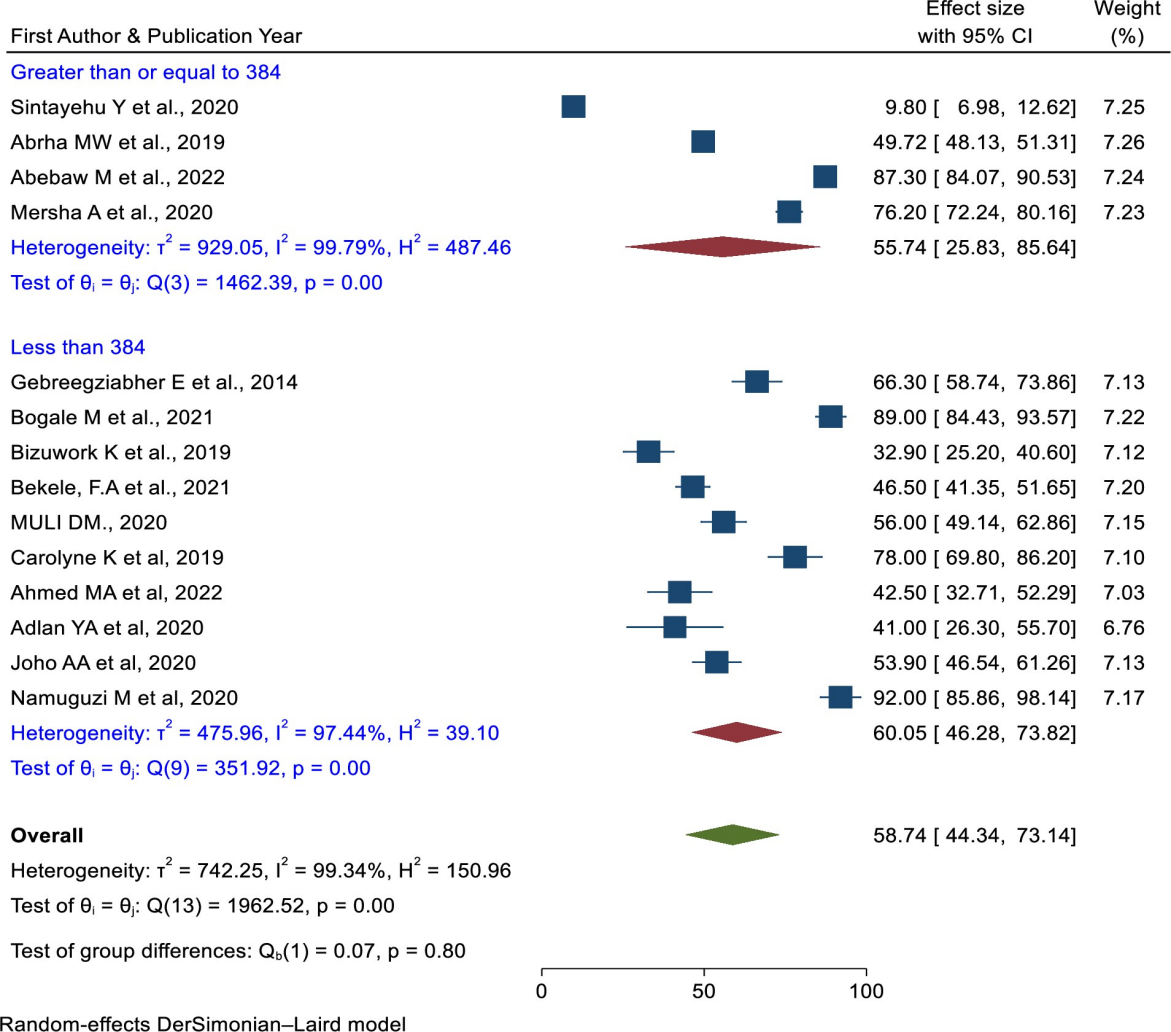
Subgroup analysis of the pooled prevalence of knowledge of newborn resuscitation among healthcare providers based on the sample size in East Africa.

Subgroup analysis was done based on the study years, and the sample sizes. Based on the study year, the prevalence of newborn resuscitation knowledge was 56.17% (95% CI = 36.43%, 75.91%) in studies conducted from 2013 to 2018, and studies conducted after 2019, the prevalence was 62.47% (95% CI = 46.66%, 78.29%) (**Fig 7**). The prevalence of knowledge on newborn resuscitation was higher in a sample size≥ 384 than in a sample size < 384 (**Fig 8**). There was significant heterogeneity in these subgroup analyses.

#### Skills of newborn resuscitation Subgroup analysis

**Fig 9:**
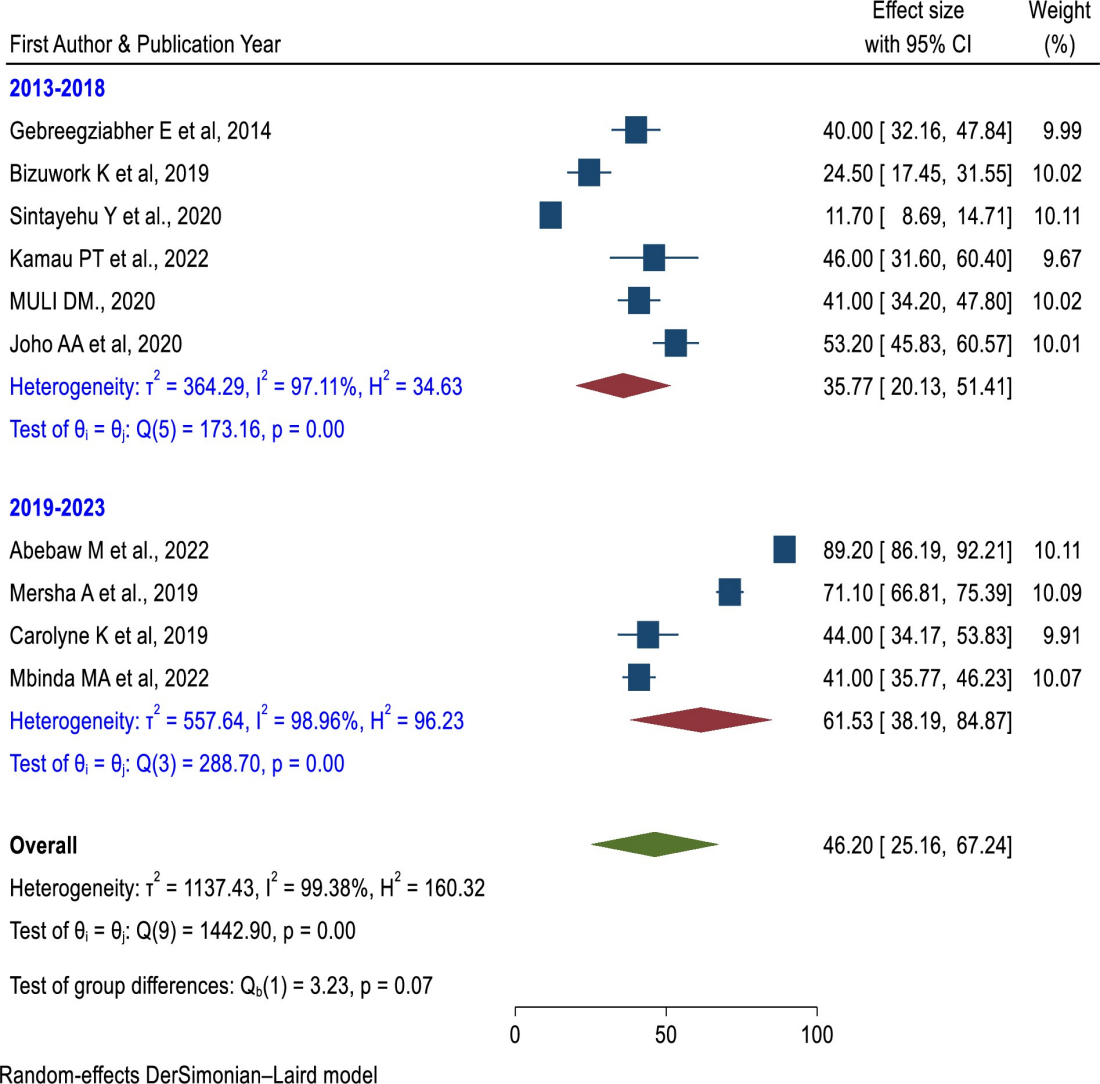
Subgroup analysis of the pooled prevalence of skills of newborn resuscitation among health care providers based on the study period in East Africa.

**Fig 10:**
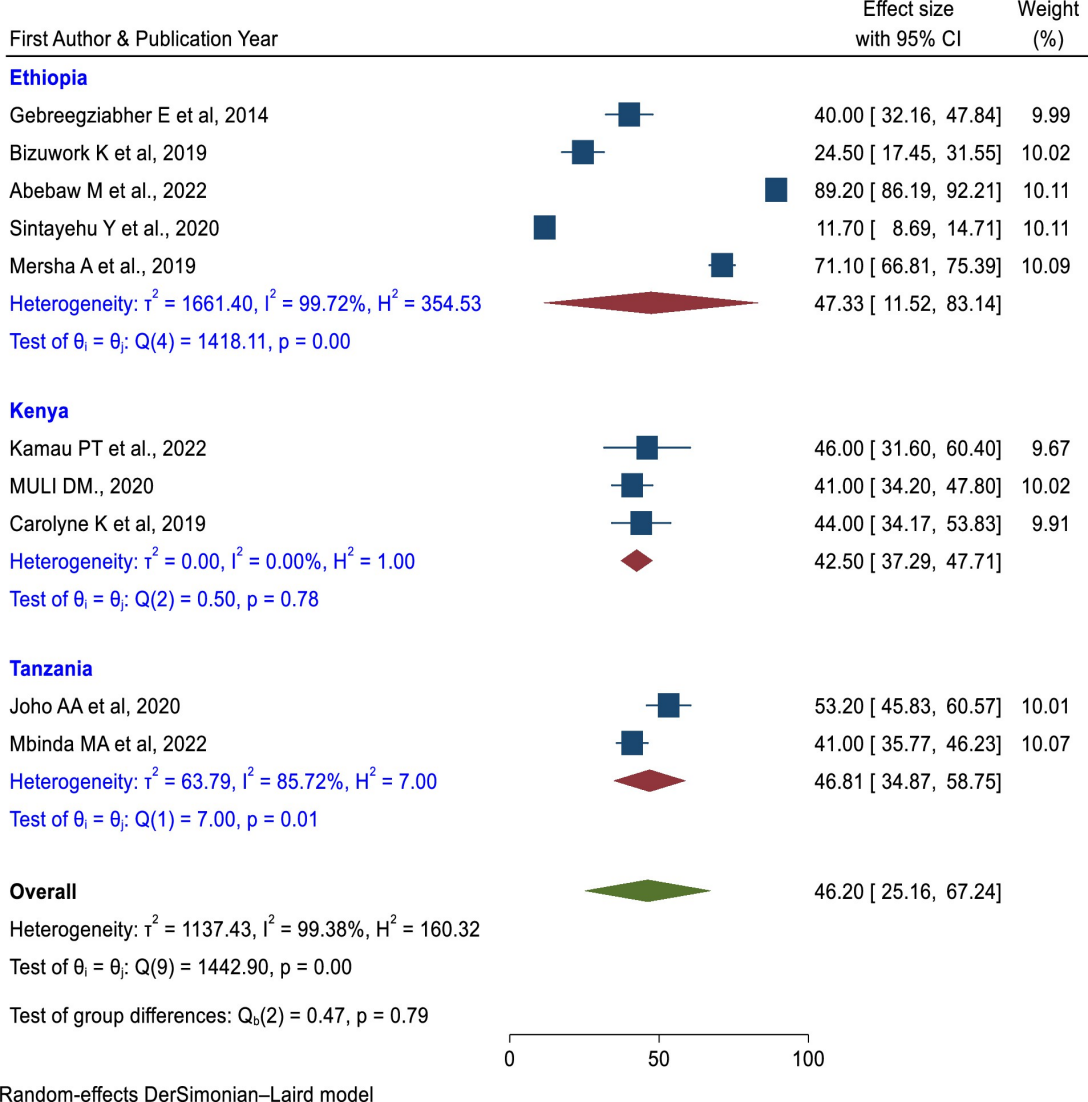
Subgroup analysis of the pooled prevalence of skills of newborn resuscitation among health care providers based on the study area in East Africa.

**Fig 11:**
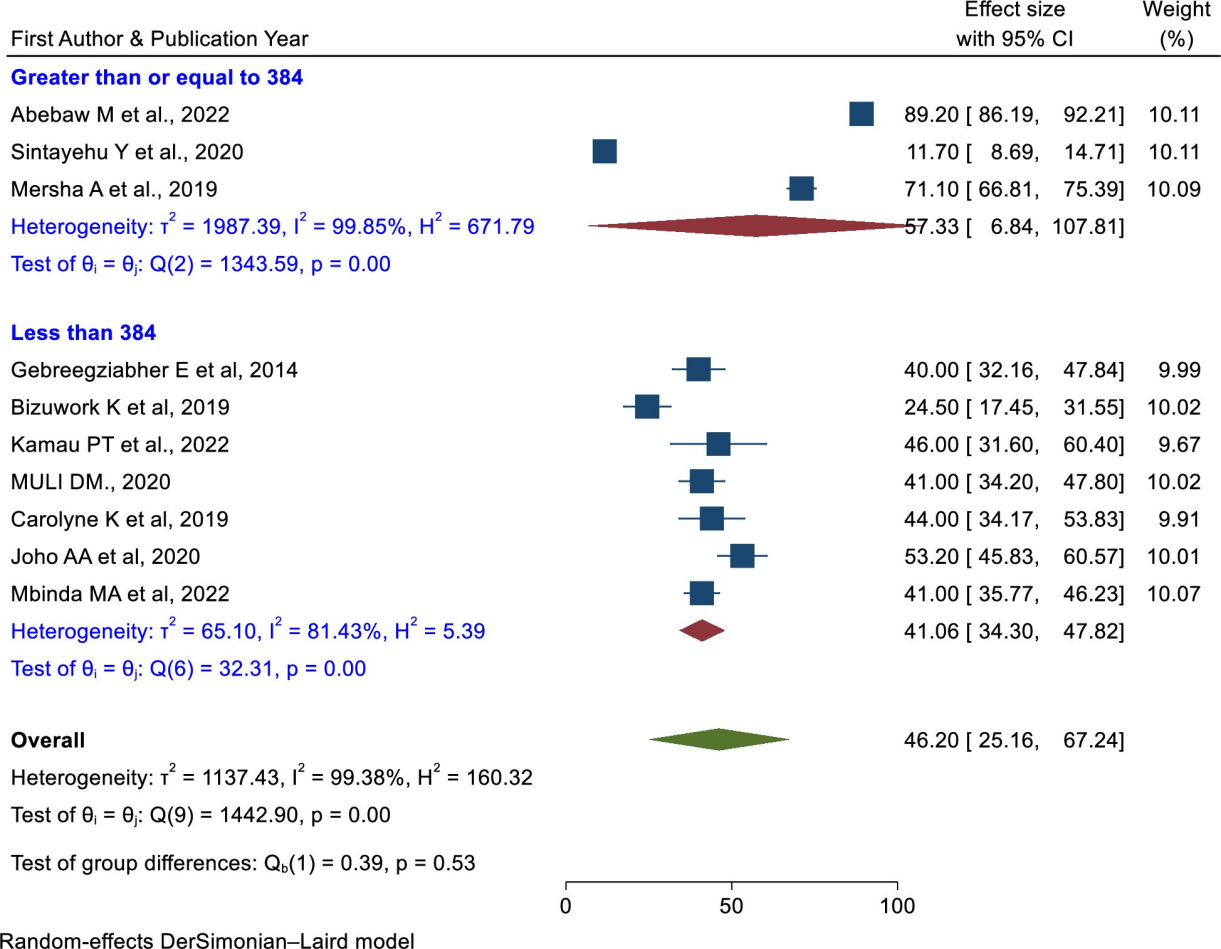
Subgroup analysis of the pooled prevalence of skills of newborn resuscitation among health care providers based on the sample size in East Africa.

In terms of the study period, there was a higher pooled prevalence of skill of newborn resuscitation among health care providers (61.53%; 95% CI: 38.19%, 84.87%) in the studies conducted after 2019 than in the studies conducted from 2013–2018 (35.77%; 95% CI: 20.13%, 51.41%) (**Fig 9**). Both categories have considerable heterogeneity. Based on the study area (Ethiopia, Kenya, and Tanzania), Kenya had the lowest prevalence (42.50%; 95% CI: 37.29%, 47.71%) and had no heterogeneity (**Fig 10**). The prevalence of newborn resuscitation skills was higher in a greater sample size (≥ 384) than in a sample size of less than 384 (**Fig 11**).

### Sensitivity analysis

**Fig 12:**
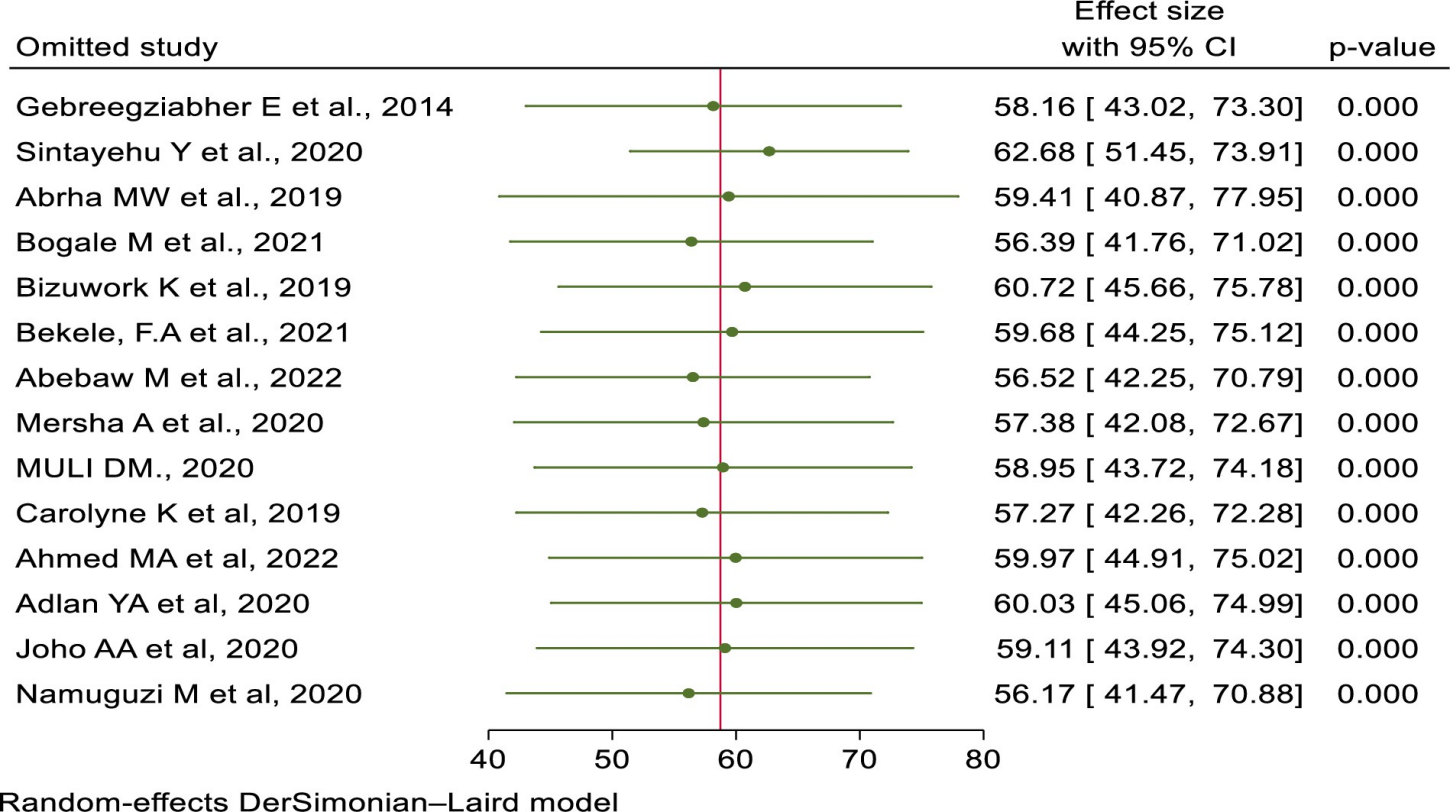
Sensitivity analyses for knowledge of newborn resuscitation and associated factors among healthcare providers in East Africa.

**Fig 13:**
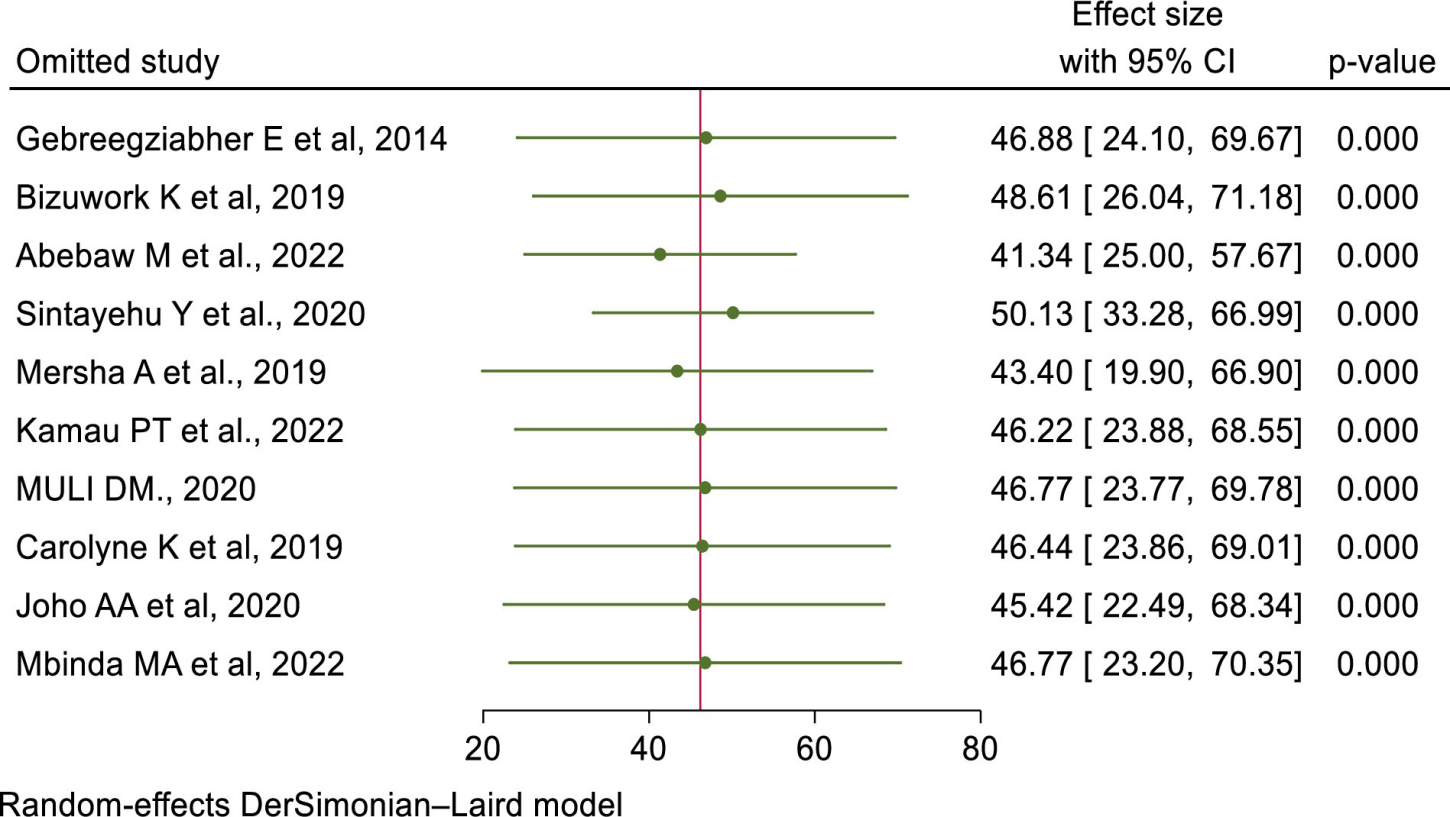
Sensitivity analyses for skills of newborn resuscitation and associated factors among healthcare providers in East Africa.

The authors have conducted a sensitivity analysis to identify the potential source of heterogeneity. Based on the results of a random-effects model, no single study affected the overall pooled prevalence of healthcare providers’ knowledge and skills in newborn resuscitation (**Figs 12 and 13**).

#### Factors associated with knowledge of newborn resuscitation

**Fig 14:**
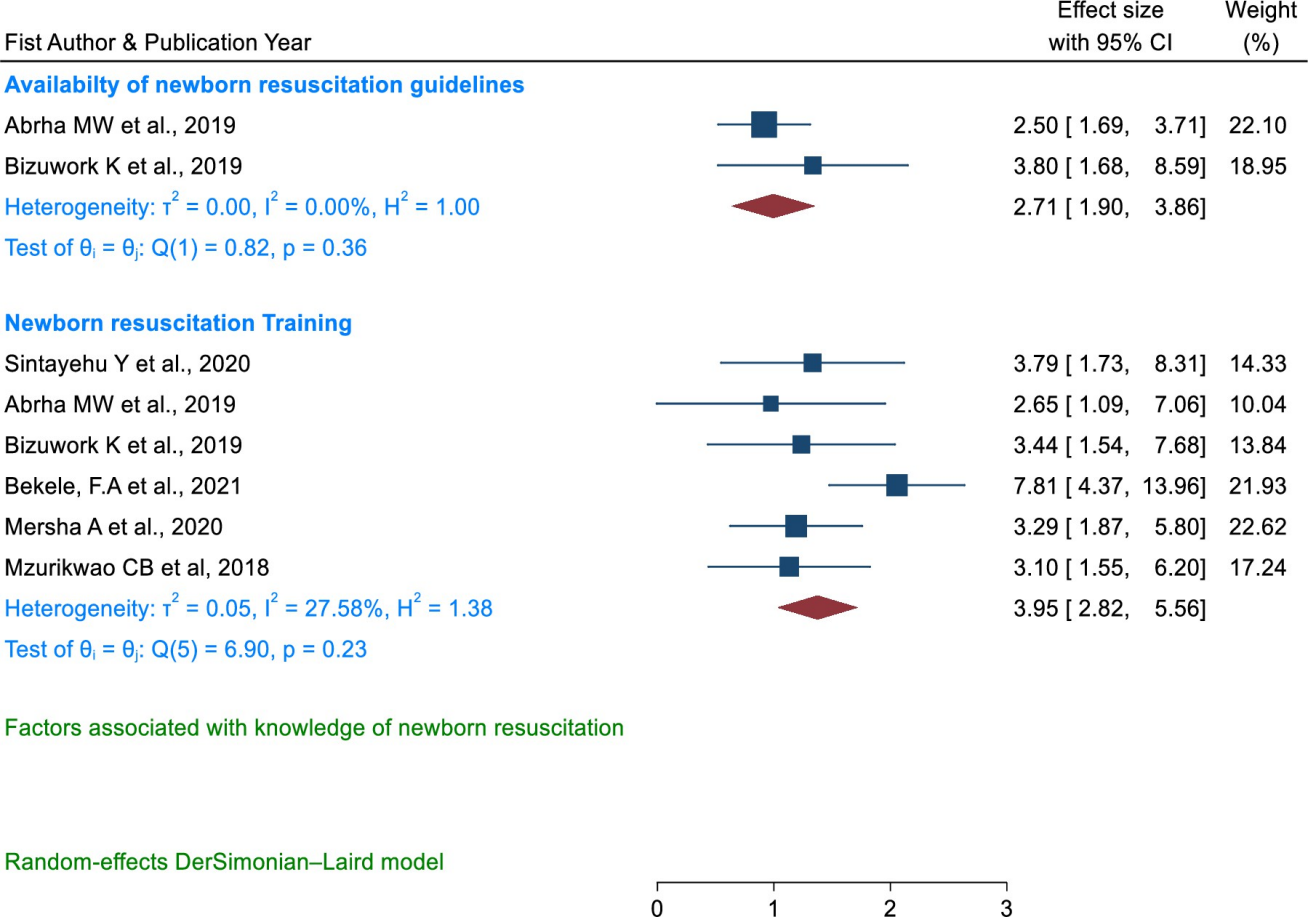
Forest plot of the association between availability of newborn resuscitation guidelines and training with knowledge of newborn resuscitation among health care providers in East Africa.

Newborn resuscitation training [7,22,45,48,49,54] and the availability of resuscitation guidelines [22,45] were associated with knowledge of newborn resuscitation. Health care providers who had taken newborn resuscitation (OR = 3.95, 95% CI: 2.82, 5.56) and the availability of newborn resuscitation guidelines in the health facilities (OR = 2.71, 95% CI: 1.90, 3.86) were associated with knowledge of newborn resuscitation (**Fig 14**).

### Factors associated with skills of newborn resuscitation

**Fig 15:**
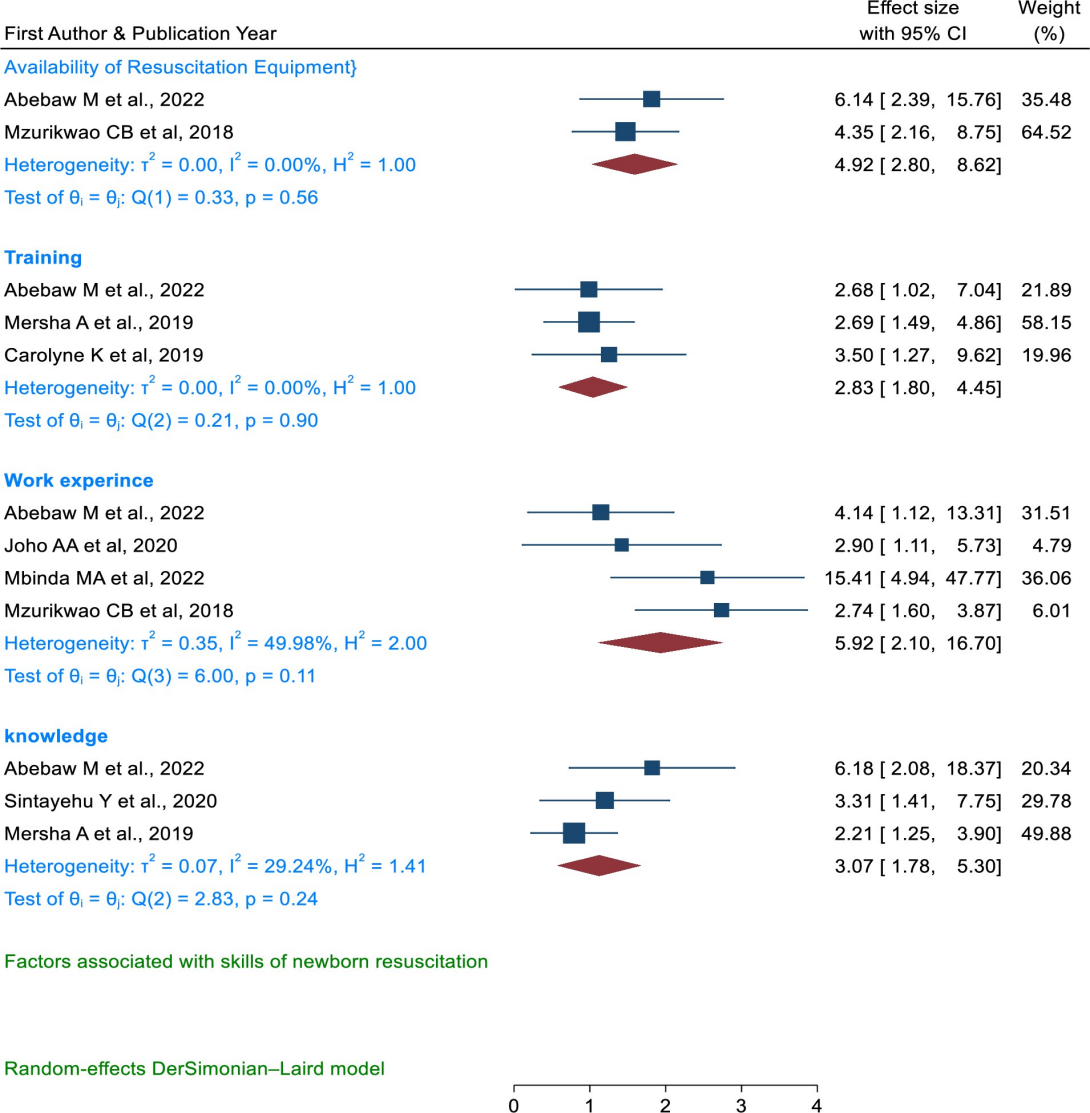
Forest plot of the association between availability of newborn resuscitation equipment, training, work experience, and knowledge with skills of newborn resuscitation among health care providers in East Africa.

Work experience, newborn resuscitation training, knowledge, and availability of newborn resuscitation equipment were associated with the skills of newborn resuscitation (**Fig 15**). Healthcare providers with more than one year of work experience (OR = 5.92, 95% CI, 2.10, 16.70) [36,53–55] were more likely to have skills in newborn resuscitation. Healthcare providers who have received training were a contributing factor for the acquisition of newborn resuscitation skills (OR = 2.83, 95%CI, 1.8, 4.45) [36,49,51]. Participants who had good knowledge about newborn resuscitation were more likely to have good skills in newborn resuscitation (OR = 3.05, 95%CI, 1.78, 5.30) [7,36,49]. The availability of newborn resuscitation equipment in the health facility was also associated with newborn resuscitation skills (OR = 4.92, 95% CI, 2.80, 8.62) [36,54].

## Discussion

Sustainable Development Goal 3 states that, by 2030, all countries will aim to reduce neonatal mortality to at least 12 per 1,000 live births and under-five mortality to at least 25 per 1,000 live births [57]. And implementing quality of care is recognized internationally as a critical aspect of the unfinished maternal and newborn health agenda, mainly concerning care during birth and in the immediate postnatal period [58]. Integrating newborn resuscitation knowledge and skills into clinical practice reduces neonatal mortality dramatically [59,60]. Therefore, to achieve this global agenda and quality of care, ensuring knowledge and skilled healthcare providers in newborn resuscitation is a key strategy [61]. The objective of this study was to assess the pooled prevalence of knowledge and skills of newborn resuscitation and the determinant factors among healthcare providers in East Africa.

The pooled prevalence of knowledge of newborn resuscitation among healthcare providers was 58.74% (95% CI: 44.34%, 73.14%). This finding was lower than the study conducted in western Nigeria [62] and Nepal [63] but greater than another study conducted in Nigeria [64]. This difference might be due to the study design and study period. In subgroup analysis, the sample size of less than 384 had a slightly higher prevalence of knowledge of newborn resuscitation (60.05%) than the sample size greater than 384 (55.74%). Based on the five-year category, the pooled prevalence of knowledge of newborn resuscitation among studies conducted between 2019 and 2023 was higher than studies conducted between 2013 and 2018. This might be because most countries have now implemented newborn resuscitation training.

The availability of newborn resuscitation guidelines in health facilities was statistically associated with healthcare provider knowledge of newborn resuscitation when compared to health facilities that did not have guidelines. This might be due to the accessibility of resuscitation guidelines at health institutions, which probably encourages healthcare providers to use them as a source of information and evidence.

The odds of knowledge of newborn resuscitation were high in participants who had taken newborn resuscitation training. A healthcare provider who had taken newborn resuscitation training was 3.95 times more likely to know participants who had not taken the training. Knowledge is recognized as one component of quality of care [65]. Evidence indicates that providing training for health care providers helps to develop the behaviors, attitudes, skills, and knowledge required to provide high quality, person-centered care. It enables them to take the right steps required to minimize risk and prepares them to respond appropriately if a risk occurs while providing care [66].

The pooled prevalence of skills of newborn resuscitation among health care providers was 46.20% (95% CI: 25.16%, 67.24%). This finding was greater than the study conducted in Nepal [63] but significantly lower than that of the study conducted in Iran [67]. The disparity might be related to differences in the magnitude and quality of newborn resuscitation training, sufficient exposure to actual resuscitation events, and health facility resuscitation setup.

In subgroup analysis, studies conducted from 2013 to 2018 had a lower pooled prevalence of the practice of newborn resuscitation (35.77%) than the studies conducted from 2019 to 2023 (61.53%). Currently, the reduction of neonatal mortality is a global agenda and one component of sustainable development goals. Due to this, newborn resuscitation training has been more advanced in the past few decades than in the previous era. Implementation of newborn resuscitation is a proven technique that increases the healthcare provider’s knowledge and skills. In addition to, the sample size subgroup analysis, the sample size greater than 384 had a higher pooled prevalence of neonatal resuscitation skills (57.33%) than the sample size less than 384 (41.06%).

One of the determinants of newborn resuscitation practice/skill among healthcare providers was the availability of newborn resuscitation equipment in health facilities. Healthcare providers who work in health facilities that had resuscitation equipment were five times more likely to have good newborn resuscitation skills than those who work in health facilities that had no resuscitation equipment. This finding was supported by a study conducted in Afghanistan [68].

The World Health Organization recommends, when a birth is anticipated, arranging delivery equipment and supplies, including neonatal resuscitation equipment [69]. The presence of equipment in health facilities may assist the healthcare professional in gaining the appropriate skill because it may help to practice day-to-day clinical practice. Moreover, the availability of equipment might encourage healthcare providers to engage in simulation-based practice. The evidence indicates that a lack of essential equipment was one barrier to performing effective neonatal resuscitation [70].

Healthcare providers who had taken newborn resuscitation training before the primary survey were 2.8 times more likely to have skills in newborn resuscitation as compared to those who had not taken the training. This finding was supported by a study conducted in Afghanistan [68] and Nepal [63]. As the World Health Organization’s recommendation, the expansion of newborn resuscitation training for healthcare providers helps to reduce neonatal mortality and strengthen the country’s health systems in general [66]. Other evidence showed that the availability of essential supplies and equipment, expanding and strengthening competency-based pre-service and in-service training was the most effective method for increasing the capacity of skilled health professionals to perform newborn resuscitation [71]. Training is essential to improve the knowledge, skills, competence, and confidence of healthcare providers in performing newborn resuscitation.

Healthcare providers who had more than one year of work experience were 5.92 times more likely to have newborn resuscitation skills than those who had less than one year of experience. This study was supplemented with a study conducted in Nepal [63]. Work experience might be an excellent way to learn about the skills of health care in clinical settings. It can also help health professionals learn more about the skills required for a wide range of roles and give those ideas about how to improve their skills or careers or where to go next.

Healthcare providers who had good knowledge of newborn resuscitation were three times more likely to have good resuscitation skills as compared to those who had poor knowledge. This finding was similar to the study conducted in Nepal [63]. Clinical knowledge, as we know, is the primary evidence for clinical care skills such as clinical decision-making, communication skills, and performance skills. As a result, improving healthcare providers’ knowledge is vital for improving their skills.

### Strengths and limitations

This meta-analysis and systematic review were based on a thorough search and independently screened, which reduced the possibility of publication bias. All sections of the manuscript were written based on the PRISM guidelines, and the quality of each study was assessed using the Newcastle-Ottawa Scale quality assessment tool. Although we found many studies to assess newborn resuscitation knowledge and skills, we were unable to obtain studies from all countries, which may have affected its representativeness.

## Conclusion

The prevalence of good knowledge and skills among healthcare providers in East Africa were low. Newborn resuscitation training and the availability of resuscitation equipment were determinant factors of both knowledge and skills of newborn resuscitation, whereas work experience, knowledge, and the availability of newborn resuscitation guidelines were associated with skills of newborn resuscitation. Newborn resuscitation training, the availability of resuscitation guidelines and equipment, and work experience are recommended to improve healthcare providers’ knowledge and skills.

## Supporting information

S1 Checklist PRISMA 2020 checklist. (PDF)

S1 File A searching strategy for knowledge and skills of newborn resuscitation among healthcare providers in East Africa, 2023. (DOCX)

S2 File Newcastle-Ottawa Quality Assessment Scale to assess knowledge and skill of newborn resuscitation among health care providers in East Africa, 2023. (DOCX)
