# Supplementary material for: Knowledge and skills of newborn resuscitation among health care professionals in East Africa. A systematic review and meta-analysis
Source: PLoS One. 2024 Mar 8;19(3):e0290737. doi: 10.1371/journal.pone.0290737 (PMC10923462; doi:10.1371/journal.pone.0290737)
Supplement: S1 File — (DOCX) [file pone.0290737.s002.docx]

Supplementary file 1 **: A searching strategy for knowledge and skills of newborn resuscitation among healthcare providers in East Africa, 2023.**

| Databases &registers | Searching terms | Number of studies |
| --- | --- | --- |
| PubMed:  <https://www.ncbi.nlm.nih.gov/pmc/> | (("knowledge"[MeSH Terms] OR "knowledge"[All Fields]) AND skills[All Fields] AND ("infant, newborn"[MeSH Terms] OR ("infant"[All Fields] AND "newborn"[All Fields]) OR "newborn infant"[All Fields] OR "newborn"[All Fields]) AND ("resuscitation"[MeSH Terms] OR "resuscitation"[All Fields]) AND associated[All Fields] AND factors[All Fields] AND ("health personnel"[MeSH Terms] OR ("health"[All Fields] AND "personnel"[All Fields]) OR "health personnel"[All Fields] OR ("health"[All Fields] AND "care"[All Fields] AND "providers"[All Fields]) OR "health care providers"[All Fields])) AND ("2013/01/01"[PubDate] : "2023/01/01"[PubDate]) | 921 |
| Google Scholar  (From January 1, 2013 to January 1, 2023 studies) | "Knowledge" AND ("skill" OR "practice") AND ("newborn resuscitation") AND (associated factors OR determinant factors) AND (health professionals OR health care providers OR health care workers) AND (Ethiopia OR Kenya OR Somalia OR Eritrea OR Djibouti OR Tanzania OR Uganda OR Rwanda OR Burundi OR South Sudan OR Sudan) | 992 |
| HINARI:  <https://login.research4life.org/tacgw/AppPortal/AppStart.cshtml?appId=3CF8651804C64F27645010F8BC6BDE2D6D636AAF&portal=1&param0=&param1=false> | Health care professionals' knowledge and skill on newborn resuscitation and associated factors (01/01/2013-01/01/2023 | 40 |
| Total retrieved |  | 1953 |
| Included |  | 17 |
