# Supplementary material for: Knowledge and skills of newborn resuscitation among health care professionals in East Africa. A systematic review and meta-analysis
Source: PLoS One. 2024 Mar 8;19(3):e0290737. doi: 10.1371/journal.pone.0290737 (PMC10923462; doi:10.1371/journal.pone.0290737)
Supplement: S2 File — (DOCX) [file pone.0290737.s003.docx]

**Supplementary file 2:** Newcastle-Ottawa Quality Assessment Scale to assess knowledge and skill of newborn resuscitation among health care providers in East Africa, 2023.

| First Author &  Author’s year | Representativeness | Sample size | None-responders | Ascertainment | Comparability | Outcome | Quality score |
| --- | --- | --- | --- | --- | --- | --- | --- |
| Gebreegziabher E et al. 2014 | 1 | 1 | 1 | 2 | 2 | 1 | 8 |
| Sintayehu Y et al., 2020 | 2 | 1 | 1 | 2 | 1 | 1 | 8 |
| Abrha MW et al., 2019 | 2 | 1 | 1 | 2 | 2 | 1 | 9 |
| Bogale M et al., 2021 | 1 | 1 | 1 | 1 | 1 | 1 | 7 |
| Bizuwork K et al., 2019 | 2 | 1 | 1 | 2 | 1 | 1 | 8 |
| Bekele, F.A et al., 2021 | 1 | 2 | 1 | 1 | 2 | 1 | 8 |
| Abebaw M et al., 2022 | 1 | 1 | 1 | 2 | 1 | 1 | 8 |
| Mersha A et al., 2020 | 1 | 2 | 1 | 1 | 2 | 1 | 8 |
| MULI DM., 2020 | 2 | 2 | 1 | 1 | 1 | 1 | 8 |
| Carolyne K et al, 2019 | 2 | 1 | 1 | 1 | 2 | 1 | 8 |
| Kamau PT et al.,2022 | 1 | 1 | 1 | 2 | 1 | 1 | 7 |
| Ahmed MA et al., 2022 | 2 | 1 | 1 | 2 | 1 | 1 | 8 |
| Adlan YA et al, 2020 | 1 | 1 | 1 | 1 | 1 | 1 | 7 |
| Joho AA et al., 2020 | 2 | 1 | 1 | 1 | 1 | 1 | 7 |
| Mzurikwao CB et al. 2018 | 1 | 2 | 1 | 1 | 2 | 1 | 8 |
| Mbinda MA et al., 2022 | 1 | 2 | 1 | 1 | 2 | 1 | 8 |
| Namuguzi M et al., 2020 | 1 | 1 | 1 | 2 | 1 | 1 | 8 |

Very Good Studies: 9-10 points, Good Studies: 7-8 points, Satisfactory Studies: 5-6 points, Unsatisfactory Studies: 0 to 4 points
